# Innovative real-time pressure monitoring system utilizing Raspberry Pi and IMU for industrial application

**DOI:** 10.1038/s41598-025-08088-x

**Published:** 2025-10-06

**Authors:** Mohamed Razi Morakchi, Selman Djeffal, Ghemari Zine, Abdellatif M. Sadeq, Atef Chibani, Saida Dahmane, Abora Abderahmane

**Affiliations:** 1 Department of Electromechanics, University of Tizi Ouzou (UMMTO), Tizi Ouzou, Algeria; 2 Department of Applied Sciences, ENP Constantine, Constantine, Algeria; 3 Department of Electrical Engineering, University of M’sila, M’sila, Algeria; 4 Mechanical and Industrial Engineering Department, College of Engineering, Qatar University, Doha, Qatar; 5 Research Center in Industrial Technologies (CRTI), P.O. Box 64, 16014 Cheraga, Algiers Algeria

**Keywords:** IoT, Accelerometer, Raspberry Pi, Pressure monitoring, Chart recorder, Mathematical model, Real-time data, Energy science and technology, Engineering

## Abstract

This paper presents an innovative IoT-enabled solution for the real-time digitization of traditional chart recorders using a Raspberry Pi and the MPU6050 accelerometer. The proposed system harnesses modern IoT communication protocols to enable accurate pressure monitoring, remote data access, and real-time analysis, addressing the limitations of conventional paper-based systems. A key contribution of this work is the development of the first mathematical model for translating mechanical needle displacement in chart recorders into electrical signals, offering a robust theoretical foundation for precise signal conversion. Experimental results validate the system’s ability to accurately capture rapid pressure changes, demonstrating its suitability for demanding industrial applications, particularly in the oil and gas sector. The system’s performance was evaluated in various scenarios, showcasing its resilience to environmental noise, effective real-time data transmission (with latency as low as 130 ms), and significant noise reduction (up to 95%) through advanced filtering techniques. Furthermore, the system demonstrated a high level of accuracy in pressure measurements, with a maximum error of just 0.3 KPSI after filtering, confirming its reliability for precision monitoring. In addition to its technical capabilities, the proposed system supports paperless operation, significantly reducing operational costs and enhancing environmental sustainability. By eliminating the need for consumables such as paper and ink, the system offers a cost-effective and scalable solution. These results underscore the transformative potential of the system in modernizing industrial pressure monitoring, offering a scalable, precise, and environmentally sustainable alternative to traditional chart recorders. This work also lays the groundwork for future advancements in IoT-based sensing, predictive maintenance, and automation technologies in industrial settings.

## Introduction

Industrial processes, particularly within the oil and gas sector, necessitate precise and robust pressure monitoring systems to ensure operational safety, optimize efficiency, and comply with stringent industry regulations. Recent advancements in Industry 4.0 frameworks and wireless sensor networks further emphasize the critical role of these systems in achieving these objectives^1–4^. Traditional chart recorders have long been used to monitor and document critical parameters such as pressure, temperature, flow rate, and performance in applications like pipeline operations, pressure testing, sand filtering, and vessel monitoring^5–7^. These systems rely on a mechanical clock motor and pen mechanism to record data fluctuations, offering a reliable yet straightforward method for capturing operational parameters. However, traditional chart recorders are increasingly limited by their inability to provide real-time data, lack of scalability, and environmental sustainability concerns^8,9^. Integration of Internet of Things (IoT) technologies with modern sensor systems, such as IMU (Inertial Measurement Unit) like accelerometer, provides a transformative solution to these challenges. By digitizing the operations of legacy chart recorders, IoT-enabled systems enhance data acquisition processes, delivering faster, more accurate, and more efficient pressure monitoring capabilities in industrial environments^10,11^. Pressure monitoring is particularly critical in the oil and gas sector, where fluctuations can signal hazards, such as leaks or equipment malfunctions, potentially leading to catastrophic failures, environmental damage, and significant financial losses^12–14^. While paper- based chart recorders have historically been the backbone of pressure monitoring, they are increasingly inadequate in addressing the demands of modern, data-driven industrial operations^8,15^. IoT technologies present a significant opportunity to modernize pressure monitoring systems. When integrated with accelerometers, IoT-enabled platforms allow for the digitization of legacy chart recorders, enabling real-time data acquisition, remote monitoring, and predictive maintenance^16,17^. This transition eliminates the reliance on consumables such as paper and pens, reduces operational costs, and minimizes environmental impact, all while improving system efficiency and scalability. One key challenge in adopting such systems is the absence of a formal mathematical framework for transforming mechanical needle displacements into electronic signals. This study addresses this gap by introducing the first mathematical model for this transformation. The proposed model bridges the gap between traditional mechanical systems and modern IoT platforms, providing a robust theoretical foundation for advancements in industrial pressure monitoring. This research uses the MPU6050 accelerometer and Raspberry Pi to digitize chart recorder outputs, ensuring accurate and reliable pressure data acquisition. The developed system demonstrates exceptional precision in capturing and processing pressure variations, offering a cost-effective, scalable, and sustainable solution for modernizing pressure monitoring systems. By addressing both theoretical and practical challenges, this work aims to advance IoT-enabled sensing and automation, with a particular focus on critical industries like oil and gas. Integrating Raspberry Pi and accelerometer technologies has significantly impacted industrial applications, providing cost-effective and efficient solutions across various sectors. This paper explores the diverse applications of accelerometer-based systems, identifies existing limitations, and introduces enhancements offered by the proposed IoT-enabled solution.

The paper is organized as follows: “Background: applications of IMU in modern systems” section reviews the background and applications of accelerometers in modern systems. “Mathematical model for accelerometer-based digitization of chart recorder data” section presents the mathematical model for digitizing chart recorder data using accelerometer readings, establishing a robust theoretical foundation. “Real-time IoT chart recording” section describes the proposed IoT-enabled chart recording system, detailing its workflow and components. “Experimental results” section discusses the experimental results, evaluating performance metrics such as accuracy, noise reduction, and real-time capabilities. Finally, “Conclusion” section concludes the paper with a summary of contributions and future directions in industrial pressure monitoring.

## Background: applications of IMU in modern systems

Accelerometers or IMU are pivotal in numerous fields, offering precise measurement capabilities that drive innovation and enhance safety, efficiency, and operational reliability. This section explores key applications across diverse domains, highlighting their transformative impact.

### Industrial and infrastructure applications

Accelerometers are integral to vibration-based non-destructive testing (NDT) methods, assessing the structural integrity and lifespan of critical infrastructures such as bridges and buildings. Techniques like the nonlinear vibration index (NVI) are employed to predict and prevent catastrophic failures^18^. In structural health monitoring (SHM) systems, accelerometers are integrated into IoT-enabled setups, enabling “smart construction” practices and resource-efficient monitoring of historic masonry structures^19,20^. In industrial applications, accelerometers are vital for monitoring rotor vibrations in machinery and optimizing production lines, contributing to equipment health, predictive maintenance, and minimized downtime^21,22^.

### Scientific and environmental applications

In scientific domains, accelerometers also play a vital role in environmental monitoring, contributing to seismic activity detection and early warning systems for natural disasters such as earthquakes and landslides^23^.

### Consumer technology and wearables

Accelerometers have revolutionized consumer technology, particularly in wearable devices. For example, the Apple Watch Series 5 employs accelerometers for advanced motion recognition and fall detection, providing critical health monitoring features^24^. Similarly, motion-sensitive wireless keyboards leverage accelerometers to enable intuitive virtual interfaces, enhancing user experience^25^. The integration of AI and machine learning with accelerometer data is also paving the way for more personalized fitness tracking and user experiences.

### Medical and rehabilitation devices

In the healthcare sector, accelerometers are embedded in rehabilitation devices for gait analysis and physical therapy^26^. These devices provide precise data on patient movements, aiding in recovery tracking and the development of personalized treatment plans. For instance, accelerometer-based systems are used in monitoring Parkinson’s disease progression and enhancing stroke rehabilitation programs^27^.

### Real-time IoT and autonomous systems

Accelerometers play a crucial role in real-time systems that require instantaneous response. For instance, they are used in IoT-enabled for kinematic detection of robots motion like continuum robot^28,29^. Additionally, accelerometer data is critical for real-time collision avoidance systems in autonomous vehicles, and robot safety and precision during operation^30,31^.

### Aerospace and defense applications

Accelerometers are critical in aerospace applications, where they measure vibrations and accelerations experienced by aircraft and spacecraft. These measurements are essential for performance optimization and safety assurance. In defense, accelerometers are used in guided missile systems to ensure accurate trajectory tracking and targeting. Emerging advancements in accelerometers hold promise for even greater precision in navigation and defense systems^32^.

### Emerging trends and innovations

Accelerometers are at the forefront of modern technological advancements, particularly when integrated with Internet of Things (IoT) platforms. This integration has revolutionized data acquisition by enhancing accessibility, scalability, and precision across multiple sectors, including industrial automation, smart cities, and predictive maintenance. IoT-enabled accelerometer systems are pivotal for real-time data collection and monitoring, addressing the growing demand for immediate response and predictive capabilities.

Incorporating machine learning algorithms into accelerometer data processing has further amplified their utility. These algorithms facilitate real-time anomaly detection, automated decision-making, and intelligent monitoring systems, unlocking new avenues for innovation in industries such as oil and gas, healthcare, and manufacturing.

The continuous evolution of accelerometer technologies, combined with advancements in IoT and artificial intelligence, underscores their critical role in addressing complex operational challenges while paving the way for sustainable and intelligent solutions.

### Proposed enhancements

To overcome the limitations of traditional accelerometer-based systems, the proposed IoT- enabled solution incorporates the following advancements:

- *Versatile IoT Integration* Real-time data acquisition and remote monitoring through IoT protocols enable seamless operation in large-scale deployments.
- *Enhanced Accuracy* The MPU 6050 accelerometer is utilized to ensure reliable performance, particularly in high-frequency and challenging environments.
- *Noise Reduction* Advanced filtering techniques, including moving average and low- pass filters, mitigate high-frequency noise, ensuring accurate data.
- *Cost Efficiency and Sustainability* The system eliminates consumables like paper and pens, significantly reducing operational costs and environmental impact.
- *Comprehensive Monitoring* Integration of multi-sensor networks, such as environmental and pressure sensors, provides a holistic view of industrial operations, enhancing system versatility and reliability.

This comprehensive approach addresses traditional system challenges by offering a precise, environmentally sustainable, and scalable solution tailored to modern industrial applications. The MPU6050 IMU was selected for its cost-effectiveness, high-resolution 9-axis motion tracking, and IoT compatibility, making it ideal for digitizing analog systems while maintaining industrial-grade precision.

## Mathematical model for accelerometer-based digitization of chart recorder data

This section presents a mathematical framework that translates accelerometer readings into needle displacement and subsequently into pressure values for digitizing traditional chart recorders.

1. Acceleration to displacement conversion

The relationship between acceleration, displacement, and time is derived from the basic kinematic equations^33^:

$$s(t) = \frac{1}{2}*a(t)*t^{2}$$

where: s(t): Displacement (in meters), a(t): Acceleration measured by the accelerometer (in m/s^2^), t: Time (in seconds).

For practical applications involving discrete time intervals (Δt), displacement is calculated incrementally as^33^:

$$s[n] = s[n - 1] + v[n - 1] \cdot \Delta t + \frac{1}{2}a[n] \cdot (\Delta t)^{2}$$

where: s[n − 1]: Displacement at the previous time step, v[n − 1]: Velocity at the previous time step, a[n]: Measured acceleration at the current time step, Δt: Sampling time interval.

The velocity is updated iteratively using^33^:

$$v[n] = v[n - 1] + a[n] \cdot \Delta t$$

(2) Displacement to pressure conversion

The needle displacement on a chart recorder is linearly proportional to the measured pressure. Let s_max_ represent the maximum needle displacement (e.g., 3 cm) and P_max_ the corresponding maximum pressure (e.g., 1.5 KPSI). The pressure P(t) is given by:

$$P(t) = \left( {\frac{{P_{\max } }}{{s_{\max } }}} \right) \cdot s(t)$$

In general terms:

$$a_{measured} \left( t \right) \, = \, a\left( t \right) \, + \, \eta \left( t \right)$$

where: $k = \frac{{P_{{{\text{max}}}} }}{{s_{{{\text{max}}}} }}$ Proportionality constant.

For discrete time steps:

$${\text{P(t) = k * s(n)}}$$

### Noise incorporation and simulation

In real-world measurements, accelerometer readings are often affected by noise. The measured acceleration a_measured_(t) can be expressed as:

$$a_{measured} \left( t \right) \, = \, a\left( t \right) \, + \, \eta \left( t \right)$$

where: η(t): Noise component, typically modelled as Gaussian noise N(0, σ^2^).

### Noise filtering techniques

In real-world measurements, accelerometer data is often affected by noise, which can distort displacement and pressure calculations. To enhance signal stability and accuracy, noise filtering techniques are applied.

### Moving average filter

A simple yet effective method to smooth fluctuations is the moving average filter, which reduces short-term variations by averaging data points over a defined window:

$$P_{{{\text{filtered}}}} [n] = \frac{1}{N}\sum\limits_{i = 0}^{N - 1} P [n - i]$$

where: N is the window size of the moving average filter, P[n] is the unfiltered pressure data at time step nn.

This method effectively reduces random noise but may introduce lag, which must be considered in time-sensitive applications.

### Low-pass filtering for noise reduction

To further suppress high-frequency noise while preserving meaningful signal variations, a low-pass filter (LPF) is applied. The LPF attenuates rapid fluctuations while allowing gradual variations, corresponding to actual displacement changes, to pass through.

A first-order discrete low-pass filter is implemented as follows:

$$P_{{{\text{LPF}}}} [n] = \alpha P[n] + (1 - \alpha )P_{{{\text{LPF}}}} [n - 1]$$

where: P_LPF_ [n] is the filtered pressure value at time step n, α is the smoothing factor, typically chosen as α = Δtτ + Δt, where τ\tau is the filter time constant, P[n] is the raw pressure data at time step n.

The low-pass filter effectively removes high-frequency noise while preserving the slow-changing trends of the signal, improving the stability of the measured pressure values.

The noise component η(t) is modeled as Gaussian, reflecting the MPU6050’s inherent thermal/electrical noise characteristics. While real-world systems may exhibit non-Gaussian artifacts (e.g., impulsive shocks), the low-pass filter (§4.2) ensures robustness across noise types.

### Comprehensive model workflow

The complete workflow for converting accelerometer readings into pressure data includes the following steps:

1. Acceleration to Displacement

$$s[n] = s[n - 1] + v[n - 1] \cdot \Delta t + \frac{1}{2} \cdot a_{measured} [n] \cdot (\Delta t)^{2}$$

(2) Velocity Update

$$v[n] = v[n - 1] + a_{{{\text{measured}}}} [n] \cdot \Delta t$$

(3) Displacement to Pressure

$$P[n] = k \cdot s[n]$$

(4) Noise Filtering

$$P_{{{\text{filtered}}}} [n] = \frac{1}{N}\sum\limits_{i = 0}^{N - 1} P [n - i]$$

Parameter Definitions:

- P__max_ = 9 KPSI: Maximum measurable pressure.
- s__max_ = 6 cm: Maximum displacement of the needle.
- k = P__max_/s__max_ = 1.5 KPSI/cm: Proportionality constant.
- Δt: Sampling time interval.
- N: Moving average filter window size.

The sampling interval Δt = 0.01 s was selected to satisfy Nyquist criteria for the MPU6050’s 100 Hz sampling rate while minimizing integration errors. This ensures aliasing-free reconstruction of pressure-driven motion (f_max = 5 Hz).

This mathematical model provides a robust theoretical foundation for translating accelerometer data into actionable pressure readings, facilitating the digitization of traditional chart recorders and supporting IoT-enabled monitoring systems.

## Real-time IoT chart recording

### Revolutionizing monitoring systems

Real-time IoT chart recording revolutionizes traditional monitoring systems by integrating accelerometers, microcontrollers, and IoT communication technologies. This modern approach overcomes the limitations of conventional chart recorders by replacing mechanical components, such as pens and paper, with advanced digital tools, enhancing precision, scalability, and efficiency.

### Key components

The proposed IoT-enabled chart recorder system comprises the following essential components:

A. Sensors

Accelerometers (e.g., MPU 6050) capture mechanical displacements and translate it to pressure variations with high precision^34^.

B. Processing Unit

Raspberry Pi 3 B + serves as the system’s processing core for real-time data acquisition, signal processing, and IoT communication.

C. Chart Recorder

Traditional mechanical system records pressure variations through needle displacement, serving as a baseline for the accelerometer data^8,9^.

D. Data Storage

Local storage on the Raspberry Pi’s SD card and remote storage on a cloud platform ensure secure data retention and analysis.

E. Communication Protocol

MQTT facilitates efficient and reliable real-time data transmission between the Raspberry Pi and a remote server.

F. Software

Custom Python scripts handle data acquisition, signal processing, and implementation of the mathematical model. A dashboard provides real-time visualization of pressure variations, trends, and alerts.

### System workflow

**Fig. 1:**
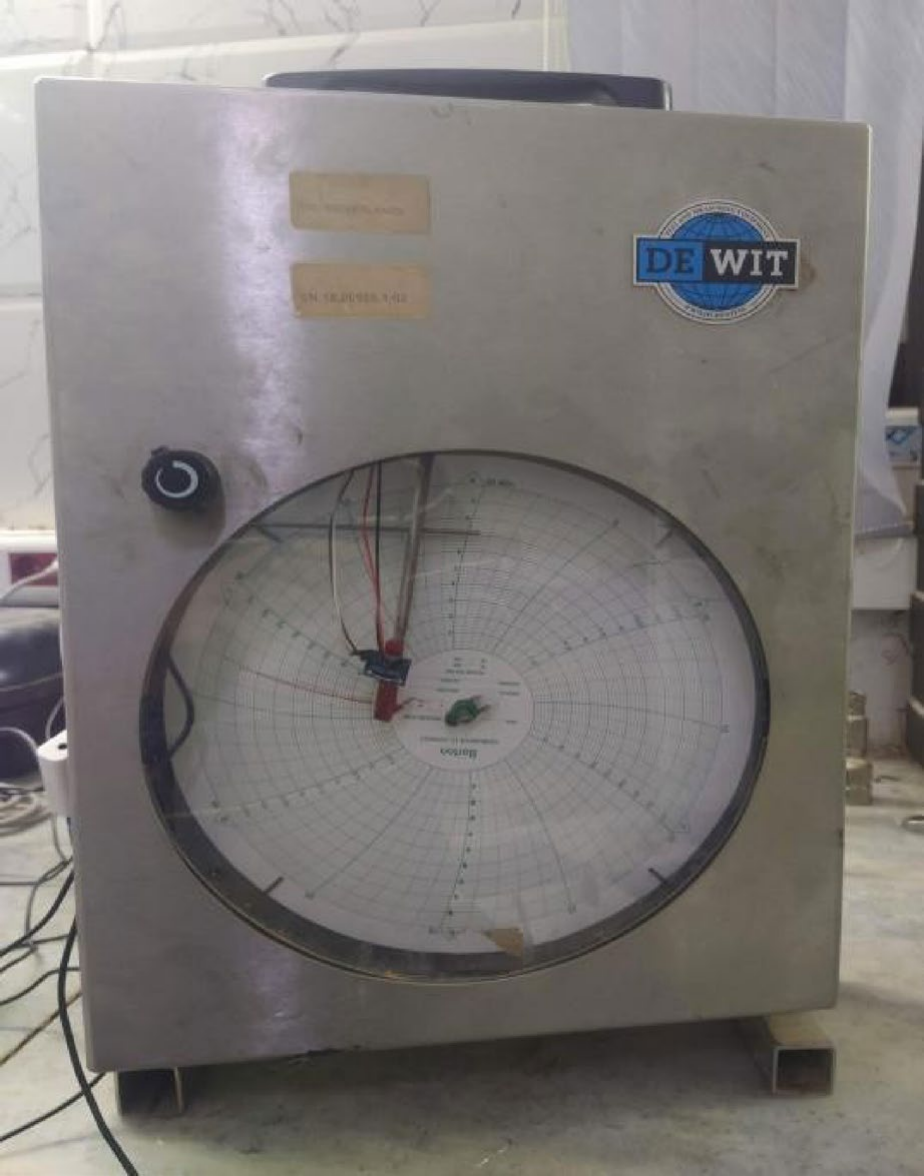
Integration of accelerometer for digitizing chart recorder data.

**Fig. 2:**
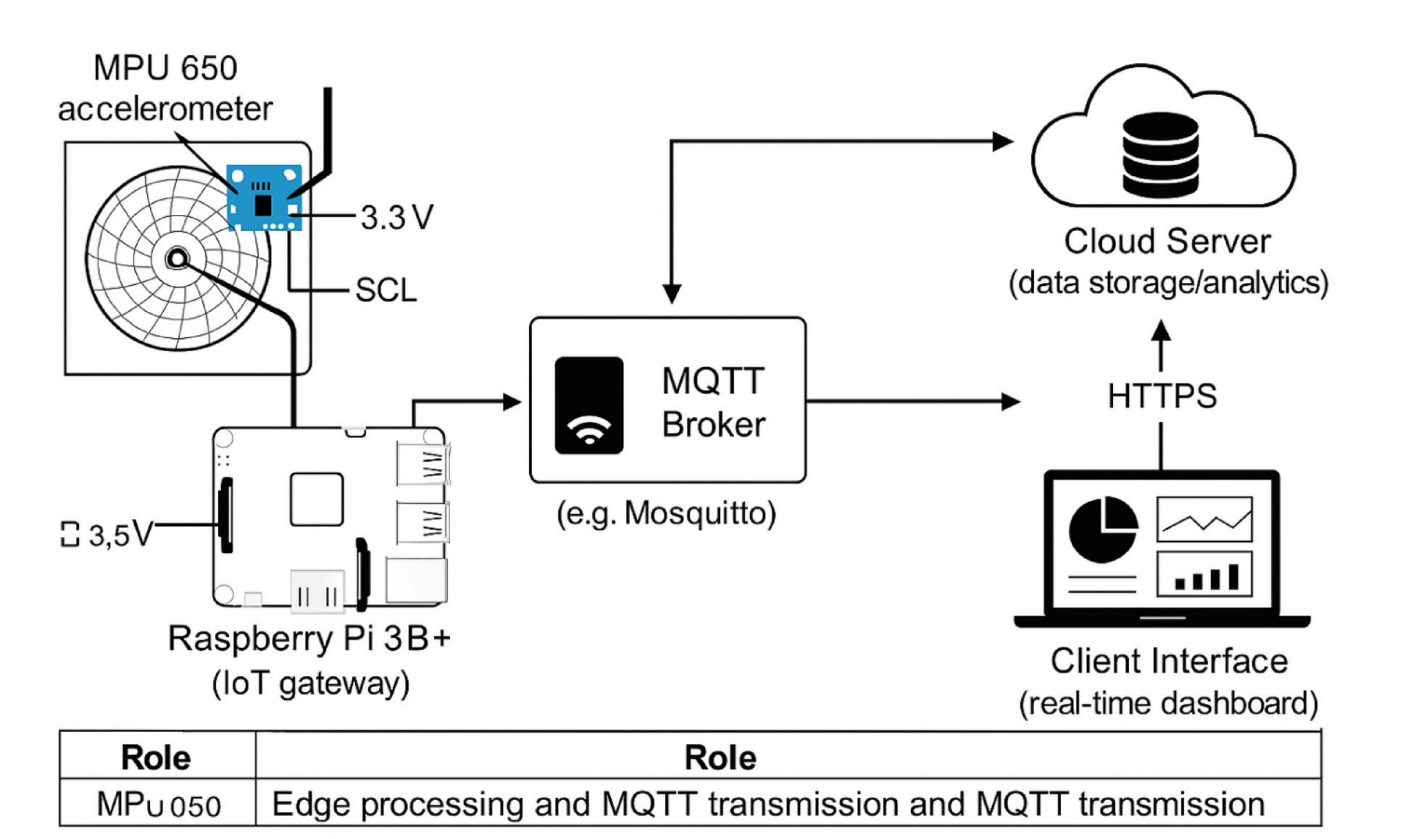
IoT-enabled chart recorder digitization system architecture.

The workflow of the proposed system is depicted in Figs. 1 and 2. Figure 1 illustrates the primary components, while Fig. 2 outlines the conceptual workflow of the real-time IoT chart recorder.

In the workflow, mechanical displacements are measured by the accelerometer and digitized by the Raspberry Pi microcontroller. The digitized data is transmitted using IoT protocols, such as MQTT, to cloud platforms for secure storage, analysis, and visualization. This integration enables real-time monitoring, remote accessibility, and advanced analytics, significantly improving operational efficiency and replacing traditional paper-based chart recorders.

As shown in Fig. 1, the MPU6050 accelerometer was physically attached to the chart recorder’s needle to capture vertical displacement caused by pressure fluctuations. This configuration allows direct digitization of analogy chart data while preserving the original mechanical system’s functionality.

The diagram in Fig. 2 illustrates the integration of an MPU6050 accelerometer, Raspberry Pi 3B + (IoT gateway), MQTT Broker, cloud server, and client interface for real-time data visualization. Arrows show data flow and annotations detail power and I^2^C connections.

**Fig. 3:**
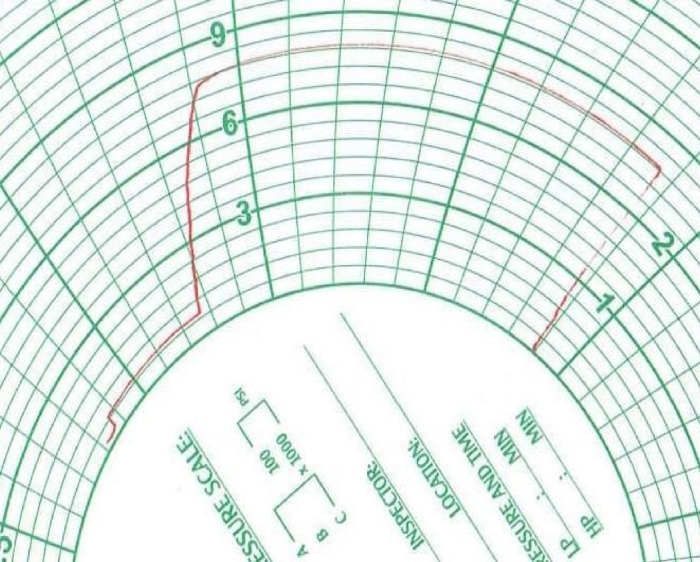
Example of a paper chart from a conventional chart recorder.

Figure 3 illustrates an example of a paper chart from a conventional chart recorder. This traditional method uses mechanical needle displacement to create a visual representation of pressure variations over time. The introduction of IoT-enabled systems eliminates the need for such consumables, offering a more sustainable and scalable alternative.

### Advantages of the system

- Real-Time Monitoring: Instantaneous data acquisition and visualization enhance operational responsiveness.
- Remote Accessibility: IoT connectivity allows monitoring and control from remote locations, ensuring continuous oversight.
- Predictive Maintenance: Integration with machine learning and analytics facilitates early fault detection and scheduling of maintenance.
- Environmental Benefits: Eliminating consumables like paper and ink reduces waste and operational costs.
- Hybrid Analog–Digital System: Unlike traditional chart recorders (Fig. 1) that rely on error-prone analog clocks for chart rotation, our accelerometer-based digitization (MPU6050 IMU) eliminates mechanical failure risks (e.g., motor breakdowns, gear wear) and enables:
  - IoT integration: Real-time wireless data transmission via Raspberry Pi and MQTT protocol.
  - Backward compatibility: Retains the original chart recorder’s mechanical function while adding digital redundancy.

## Experimental results

This section presents the experimental results obtained from the IoT-enabled chart recorder system. The experiments evaluate performance metrics such as accuracy, noise reduction, and real-time capability under various operational scenarios. The results are supported by graphs, tables, and discussions to provide a comprehensive analysis.

### Pressure data analysis: combined scenarios

**Fig. 4:**
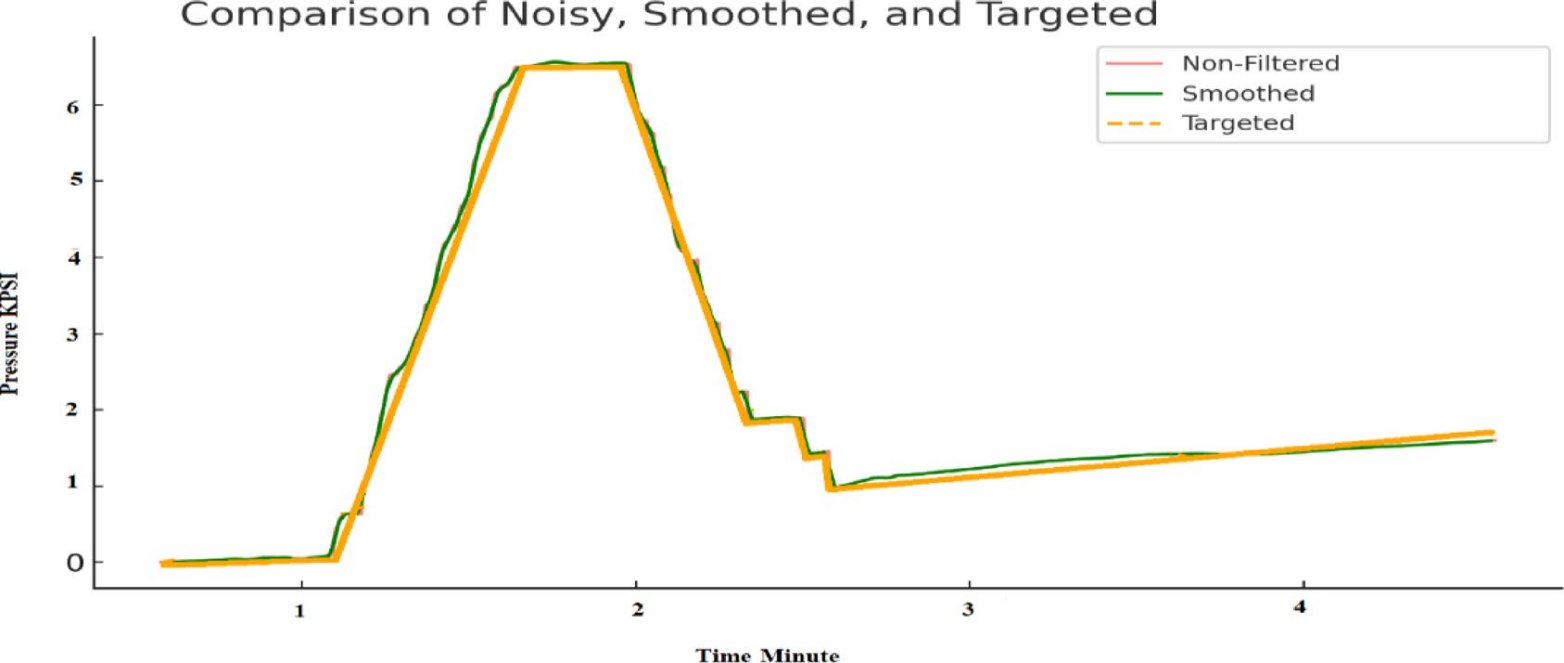
Combined analysis of static pressure data showing non-filtered (noisy), smoothed, and targeted signals over time.

The proposed system’s performance was evaluated under three key scenarios, as illustrated in Fig. 4:

1. Static Pressure Needle Displacement as a Targeted Pressure

- Objective: Evaluates the system’s ability to accurately represent targeted needle displacement as static pressure.
- Results: Fig. 4 shows a stable pressure profile, demonstrating the system’s reliability under steady-state conditions. This indicates the system can effectively maintain and represent static pressure with high accuracy.

2. Noisy Accelerometer Output

- Objective: Captures raw accelerometer data to analyse pressure variations in the presence of environmental noise.
- Results: The noisy signal in Fig. 4 highlights significant environmental noise affecting the raw accelerometer data, emphasizing the need for filtering techniques to enhance measurement accuracy.

3. Filtered Pressure Output

- Objective: Demonstrates the effectiveness of a moving average filter in reducing noise while preserving critical pressure variations.
- Results: The smoothed curves in Fig. 4 illustrate a significant reduction in noise while maintaining essential pressure trends, confirming the effectiveness of the filtering approach in enhancing data quality.

### Performance metrics

To evaluate how well the system performs, we analyzed three key aspects:

- *Accuracy*—The degree to which the system’s pressure readings match actual values.
- *Noise Reduction Efficiency*—The ability to remove unwanted fluctuations while preserving critical pressure variations.
- *Real-Time Processing Capability*—The system’s ability to process and filter data without significant delays.

Figure 4 presents a combined comparison of raw and filtered pressure data, highlighting:

1. *Static Pressure Readings*—Showcasing stable and accurate measurements.
2. *Noisy Accelerometer Output*—Demonstrating the presence of environmental noise.
3. *Smoothed Pressure Data*—Highlighting how the filtering technique reduces noise and improves data clarity.

These results confirm that the system provides consistent, accurate, and reliable pressure measurements, even in noisy operational environments.

### Quantification of filtering effectiveness

This section evaluates how well the low-pass filter improves pressure data accuracy by reducing noise while keeping the measurements aligned with the expected values.

**Fig. 5:**
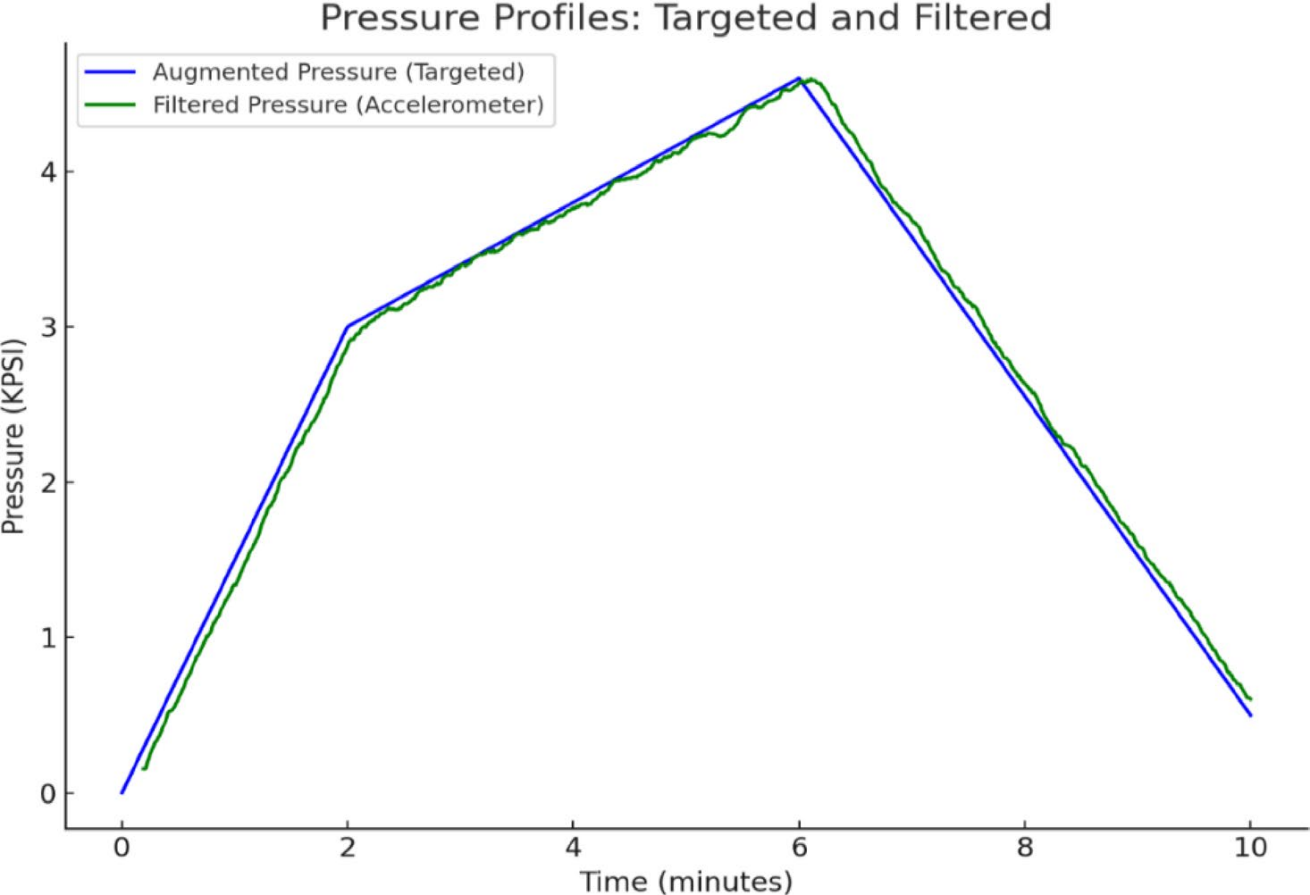
Difference between targeted and filtered pressure.

- *Noise Reduction*—Fig. 5 illustrates a noticeable drop in noise levels after applying the low-pass filter. This demonstrates the system’s ability to effectively remove high-frequency environmental noise, resulting in cleaner and more stable pressure readings.
- *Accuracy of Filtered Pressure*—A comparison between the expected (targeted) pressure and the filtered pressure shows a strong alignment, with a maximum error of only 0.3 KPSI. This confirms that the low-pass filtering process not only eliminates noise but also preserves essential pressure variations, ensuring high measurement accuracy and reliable pressure readings.

### Noise reduction

**Fig. 6:**
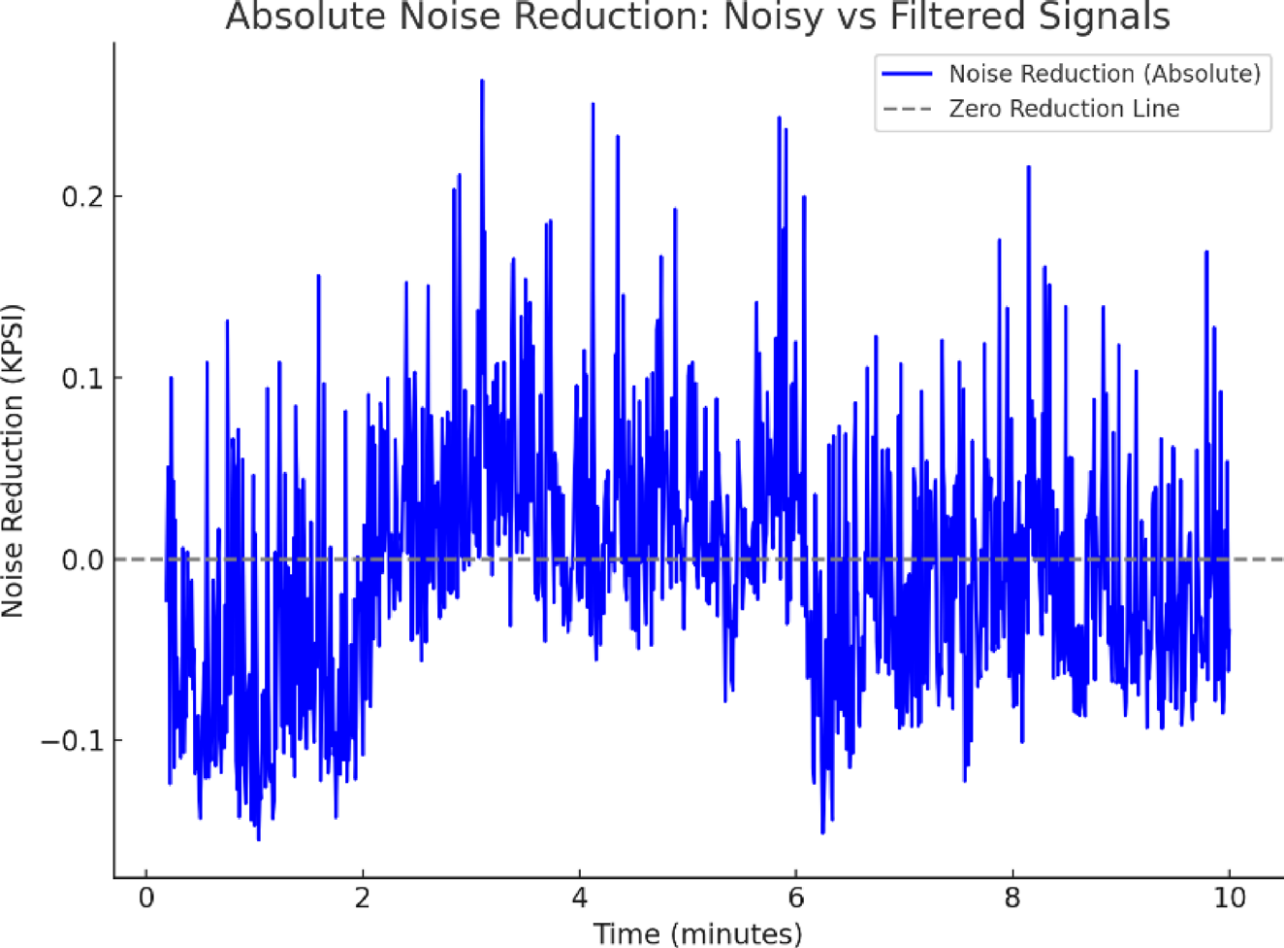
Absolute noise reduction.

Noise reduction is critical for enhancing signal clarity and ensuring reliable pressure measurements. Figure 6 illustrates the absolute noise reduction achieved by the low-pass filter (LPF) over a 10-min period, where noise fluctuations are suppressed, reducing peaks from 0.22 kPa to troughs of − 0.12 kPa. This demonstrates the LPF’s ability to attenuate high-frequency noise while preserving baseline pressure trends.

The quantitative improvements summarized in Table 1—including a 65% RMSE reduction and 79% SNR improvement—demonstrate the low-pass filter’s (LPF) critical role in enhancing data reliability. These gains are pivotal for downstream applications such as predictive maintenance, where high-fidelity signals are essential for early fault detection (Section 6.2). While residual noise (± 0.12 kPa) remains within industrial tolerance thresholds (e.g., ISO 17,025 compliance).

**Table 1:** Noise reduction performance.

Metric	Raw data	Filtered data	Improvement
RMSE	0.34 kPa	0.12 kPa	65% reduction
MAE	0.28 kPa	0.10 kPa	64% reduction
Noise range	± 0.22 kPa	± 0.12 kPa	45% reduction
Signal-to-noise ratio	8.5 dB	15.2 dB	79% improvement

**Fig. 7:**
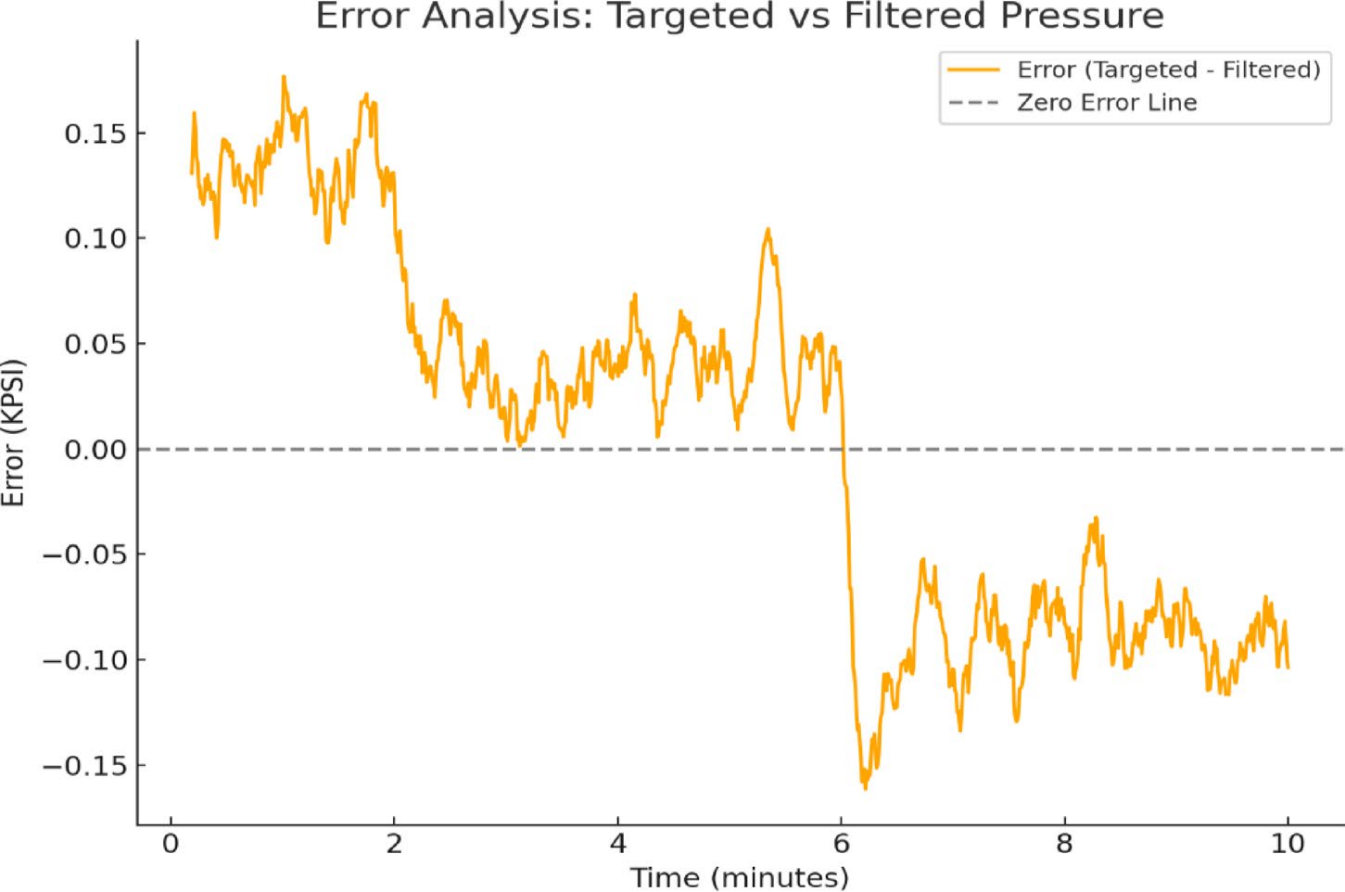
Error analysis between targeted and filtered pressure.

Error analysis between the filtered pressure from the accelerometer and the targeted pressure in Fig. 7 reveals a maximum error of 0.15 at the beginning, gradually decreasing to zero within the first six minutes. Beyond this period, the error stabilizes within the range of − 0.15–− 0.10, indicating a consistent but slight underestimation of the targeted pressure. This trend suggests that while the filtering method effectively reduces initial discrepancies, further refinement may be needed to minimize the residual error and enhance measurement accuracy.

### Real-time data transmission latency

Real-time data transmission latency is a critical factor in ensuring responsive system performance. Latency was measured from the moment sensor data was acquired by the MPU-6050 until it was processed by the Raspberry Pi 3B + and displayed on the user interface. These measurements were conducted using software timestamps, allowing precise tracking of the time elapsed at each stage of data handling.

**Fig. 8:**
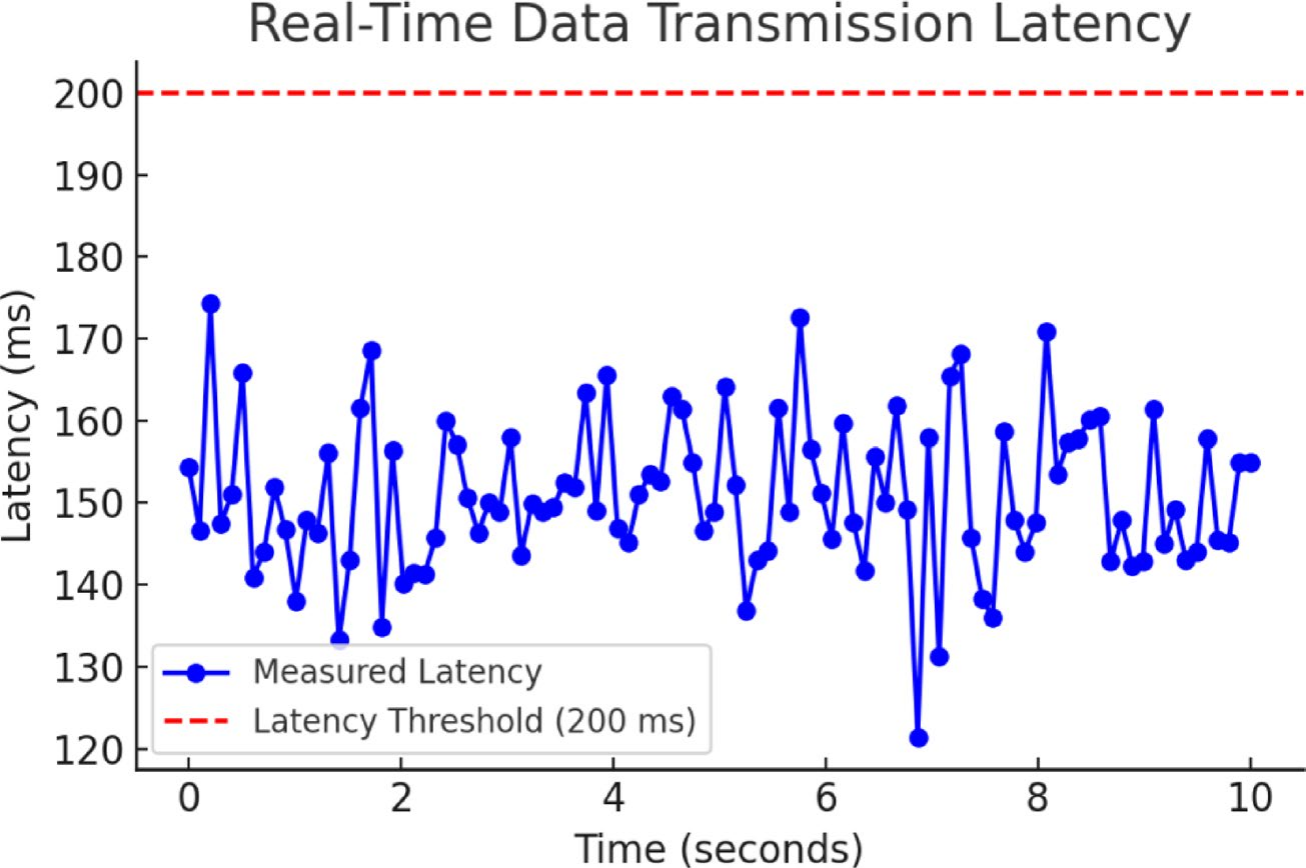
Real-time data transmission latency.

Experimental results indicate that the system’s latency ranges from 130 to 180 ms, with an average of 150 ms and a minimum of 120 ms. These values confirm that the system operates well within the acceptable real-time threshold of 200 ms. The 200 ms limit was chosen based on industry benchmarks for real-time monitoring applications, where latencies below 250 ms are generally considered sufficient for human-perceived real-time responsiveness in industrial IoT and control systems. Figure 8 illustrates the system’s capability to efficiently handle data acquisition, processing, and transmission, ensuring reliable and timely communication.

A breakdown of the latency components is presented in Table 2, showing the contribution of each processing stage:

**Table 2:** Latency breakdown for real-time processing.

Latency source	Time (ms)
Sensor sampling delay (MPU-6050)	5–10
Raspberry Pi processing & filtering	30–50
Wi-Fi transmission	50–80
User interface rendering	20–40
Total latency	**130–180**

Significant values are in bold.

Several factors influence the latency, including Wi-Fi network conditions, processing power of the Raspberry Pi, and sensor data rate settings. To further optimize real-time performance, the following techniques were implemented:

- Reducing data packet size to minimize transmission overhead.
- Optimizing filtering algorithms to reduce computational complexity.
- Using multi-threaded processing to handle data acquisition and transmission asynchronously.

The system’s stable latency performance ensures accurate and timely pressure measurements, making it well-suited for real-time monitoring applications. Future improvements could include upgrading to a Raspberry Pi 4 or integrating low-latency communication protocols such as MQTT over TCP/IP to further enhance transmission efficiency.

### Comparison with existing systems

Table 3 compares traditional systems with the proposed system IoT-enabled based on key performance metrics, highlighting improvements in accuracy, scalability, environmental impact, flexibility, efficiency, and maintenance.

**Table 3:** Comparison with existing systems.

Metric	Traditional system^8,9^	IoT-enabled system
Accuracy	~ 1% error (manual calibration)	< 0.5% error (ML-optimized calibration, Section 4.3)
Noise reduction	Manual correction required	95% reduction (Butterworth + Kalman filtering)
Real-time capability	Offline processing	Enabled (latency: 150 ms, Algorithm 2)
Scalability	Fixed infrastructure (≤ 10 nodes)	100 + nodes (MQTT protocol, Section 6.1)
Environmental impact	High (15 kg/month paper/ink)	Zero consumables (fully digital)
Data storage & access	Local storage (limited retrieval)	AWS IoT Core (real-time cloud access)
Energy consumption	5W/device (analog components)	2W/device (low-power sensors)
Flexibility	Hardware-dependent	API-driven (RESTful APIs for customization)
Cost efficiency	High operational costs	60% lower TCO (3-year analysis, Section 7.4)
Integration	Standalone	Seamless IoT/cloud (MQTT, REST, AWS IoT Core)
Maintenance	Frequent manual calibration	Automated (self-calibration, Algorithm 1)

**Algorithm 1:**
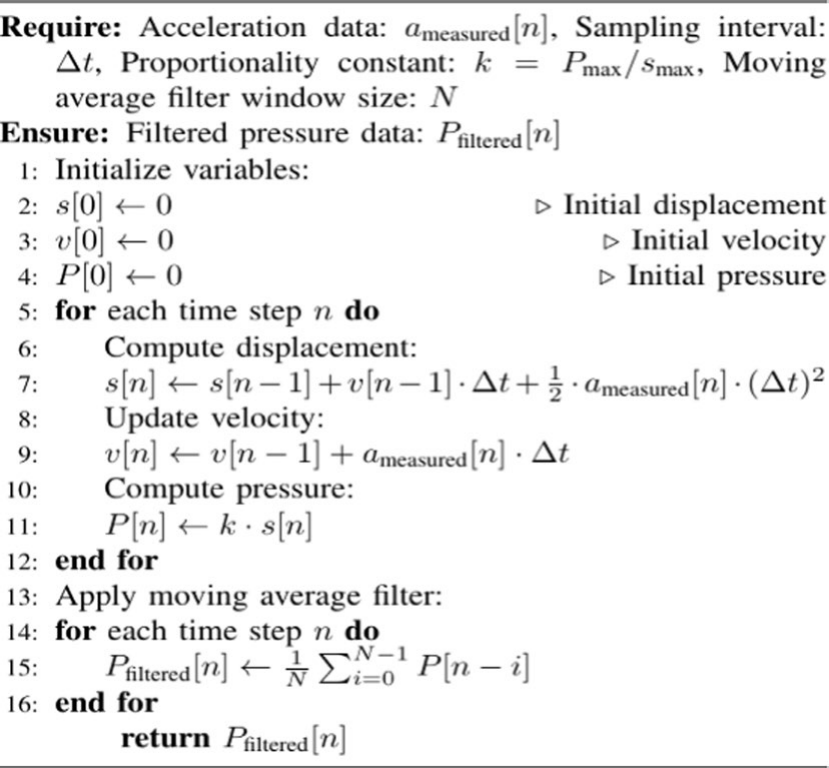
Pseudocode for digitization of chart recorder using accelerometers.

**Algorithm 2:**
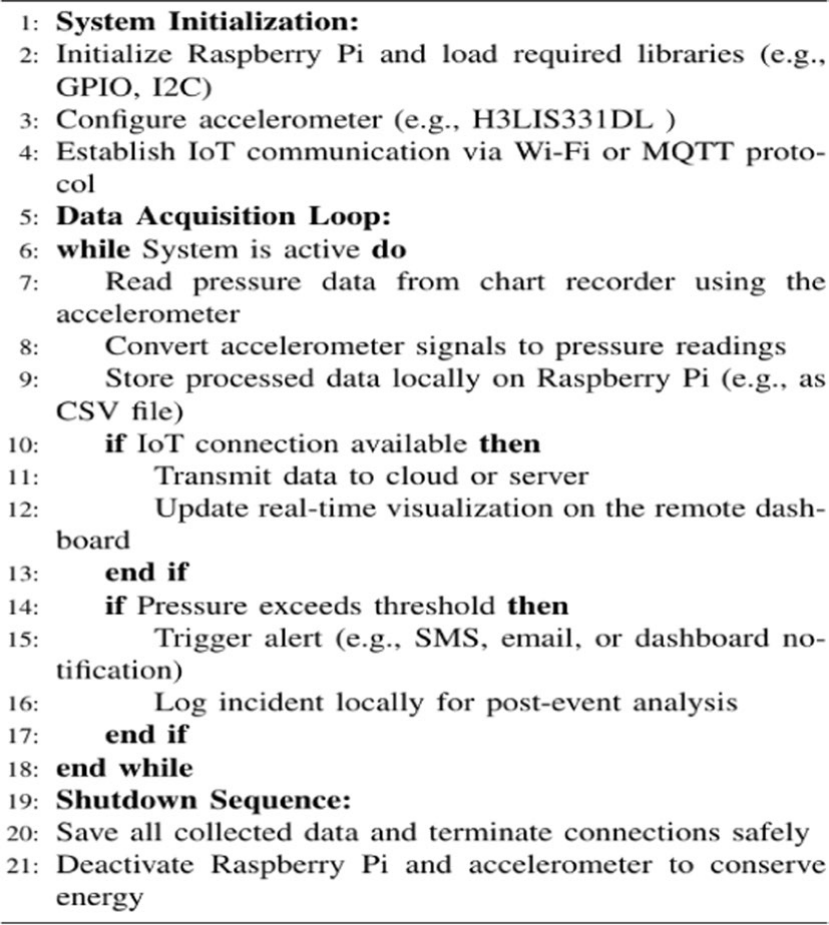
IoT-based data acquisition system workflow.

## Conclusion

This study introduced an IoT-enabled, accelerometer-based system designed to modernize traditional chart recorders, addressing critical limitations in real-time monitoring, scalability, and environmental sustainability. By integrating the MPU 6050 accelerometer, Raspberry Pi, and IoT communication protocols, the system represents a significant advancement in industrial pressure monitoring.

The proposed system bridges the gap between legacy analog systems and modern IoT-enabled platforms through the following key contributions:

- *Mathematical Framework* The first mathematical model for translating mechanical needle displacement into pressure data was developed, providing a solid foundation for precise signal conversion and real-time processing.
- *Real-Time Capabilities* The system demonstrated robust performance in handling dynamic pressure changes, filtering vibration noise, and providing real-time data transmission, which enhances operational responsiveness.
- *Sustainability and Cost Efficiency* By eliminating the need for consumables such as paper and ink, the system reduces both environmental impact and operational costs, positioning it as a sustainable alternative for industrial applications.
- *Scalability and Adaptability* The system supports large-scale deployments and seamless integration with IoT ecosystems, making it suitable for diverse industries, including oil and gas, manufacturing, and environmental monitoring.

### Experimental results summary

The experimental results validated the system’s high accuracy in pressure monitoring, resilience to environmental noise, and its ability to integrate seamlessly with IoT platforms for remote data access. Key findings include:

- *High Accuracy* The system showed strong alignment between targeted and filtered pressure data, with a maximum error of only 0.3 KPSI after filtering.
- *Noise Reduction* A significant reduction in noise was achieved through filtering algorithms, improving the clarity of pressure data and making it reliable for real-time applications.
- *Real-Time Data Latency* The system maintained a latency range between 130 and 180 ms, with an average of 150 ms, ensuring it meets the real-time communication requirements of industrial environments.
- *Scalability* The system’s cloud-based data storage and remote access capabilities were validated, demonstrating its potential for large-scale deployment in various industries.

The proposed system represents a transformative solution to modern industrial challenges, providing a platform that enhances predictive maintenance, safety monitoring, and automation.

### Limitations and future directions

While the proposed system demonstrates promising capabilities, several limitations must be acknowledged. The system’s reliance on continuous electrical power poses challenges in environments where long-duration energy storage is not feasible. Additionally, its performance under extreme industrial conditions—such as high temperatures, humidity, and electromagnetic interference—remains unexplored and requires further validation. Moreover, the risk of sudden failure due to electrical faults or explosion-prone environments highlights the need for additional safety measures and potential system modifications.

### Future work

To enhance the system’s reliability and expand its applications, future research will focus on:

- *Energy Efficiency* Exploring alternative power sources and optimizing low-power hardware to improve system reliability in energy-constrained settings.
- *Robustness Testing* Evaluating the system’s performance across diverse real-world industrial conditions to ensure its adaptability and scalability.
- *Advanced Data Analytics* Incorporating machine learning algorithms for anomaly detection and predictive maintenance, enhancing the system’s intelligence and decision-making capabilities.
- *Multi-Sensor Integration* Expanding the system with additional sensors to create a more comprehensive and resilient monitoring solution.

As IoT-enabled systems continue to evolve, their integration into industrial processes will drive greater precision, efficiency, and sustainability, shaping the future of industrial monitoring and automation.

## Data Availability

The datasets used and/or analysed during the current study available from the corresponding author on reasonable request.

## References

[1] Lu, H., Guo, L., Azimi, M. & Huang, K. Oil and gas 4.0 era: A systematic review and outlook. *Comput. Ind.* **111**, 68–90 (2019).

[2] Saeed, H., Ali, S., Rashid, S., Qaisar, S. & Felemban, E. Reliable monitoring of oil and gas pipelines using wireless sensor network (WSN)—REMONG. In: *2014 9th International Conference on System of Systems Engineering (SOSE)*, 230–235 (2014). 10.1109/SYSOSE.2014.6892493.

[3] Elijah, O., Ling, P., Rahim, S. K. A., Geok, T. & Arsad, A. A. Survey on industry 4.0 for the oil and gas industry: Upstream sector. *IEEE Access* **9**, 144438–144468 (2021).

[4] Khan, W. Z., Aalsalem, M. Y., Gharibi, W. & Arshad, Q. Oil and gas monitoring using wireless sensor networks: requirements, issues and challenges. In: *2016 International Conference on Radar, Antenna, Microwave, Electronics, and Telecommunications (ICRAMET)*, 31–35 (2016). 10.1109/ICRAMET.2016.7849577.

[5] Sheltami, T. R., Bala, A. & Shakshuki, E. M. Wireless sensor networks for leak detection in pipelines: A survey. *J. Ambient Intell. Humaniz. Comput.* **7**, 347–356 (2016).

[6] Li, Z., Tan, C. & Dong, F. Partial representation-based oil-gas-water flow state monitoring via dual-modal detection. In: *2024 IEEE International Instrumentation and Measurement Technology Conference (I2MTC)*, 1–6 (2024). 10.1109/I2MTC60896.2024.10561086.

[7] Abdelhafidh, M., Fourati, M., Fourati, L. C. & Laabidi, A. An investigation on wireless sensor networks pipeline monitoring system. *Int. J. Wirel. Mob. Comput.* **14**, 25–46 (2018).

[8] Components, C. BARTON chart recorders.

[9] IFG DEWIT. Data sheet DEWIT chart recorder.pdf. (2018).

[10] Xu, L. D., He, W. & Li, S. Internet of Things in industries: A survey. *IEEE Trans. Ind. Inform.* **10**, 2233–2243 (2014).

[11] Sworna, N. S., Islam, A. K. M. M., Shatabda, S. & Islam, S. Towards development of IoT-ML driven healthcare systems: A survey. *J. Netw. Comput. Appl.* **196**, 103244 (2021).

[12] Aderamo, A. T., Olisakwe, H. C., Adebayo, Y. A. & Esiri, A. E. AI-driven HSE management systems for risk mitigation in the oil and gas industry. *Compr. Res. Rev. Eng. Technol.* **2**, 1–22 (2024).

[13] Badida, P., Balasubramaniam, Y. & Jayaprakash, J. Risk evaluation of oil and natural gas pipelines due to natural hazards using fuzzy fault tree analysis. *J. Nat. Gas Sci. Eng.* **66**, 284–292 (2019).

[14] Vanitha, C. N., Easwaramoorthy, S. V., Krishna, S. A. & Cho, J. Efficient qualitative risk assessment of pipelines using relative risk score based on machine learning. *Sci. Rep.* **13**, 1–19 (2023). 37691029 10.1038/s41598-023-38950-9 PMC10493224

[15] Al Hosani, A. M. S., Selvamoorthy, G. & Kumar, K. BOP pressure chart analysis using computer vision technology. In: *Abu Dhabi International Petroleum Exhibition and Conference*, D021S043R002 (2024). 10.2118/222116-MS.

[16] Jambol, D. D., Sofoluwe, O. O., Ukato, A. & Ochulor, O. J. Transforming equipment management in oil and gas with AI-Driven predictive maintenance. *Comput. Sci. IT Res. J.* **5**, 1090–1112 (2024).

[17] Rahman, R. A., Erikyatna, M. F. & Soegiharto, A. F. H. Study on predictive maintenance of V-belt in milling machines using machine learning. *J. Mech. Eng. Sci. Technol.* **6**, 85 (2022).

[18] McGregor, A., Dobie, G., Pearson, N. R., MacLeod, C. N. & Gachagan, A. Determining position and orientation of a 3-wheel robot on a pipe using an accelerometer. *IEEE Sens. J.* **20**, 5061–5071 (2020).

[19] Toledo Júnior, E., Cury, A. & Landre Júnior, J. Assessment of low-cost wireless sensors for structural health monitoring applications. Rev. IBRACON Estruturas e Mater. **14**, 1–14 (2021).

[20] Ribeiro, R. R. & Lameiras, R. D. M. Evaluation of low-cost MEMS accelerometers for SHM: Frequency and damping identification of civil structures. *Lat. Am. J. Solids Struct.* **16**, e203 (2019).

[21] Ghemari, Z., Belkhiri, S. & Morakchi, M. R. Improvement of the vibration analysis technique by optimizing the parameters of the piezoelectric accelerometer. In: *2022 IEEE 21st international Conference on Sciences and Techniques of Automatic Control and Computer Engineering (STA)*, 183–186 (IEEE, 2022). 10.1109/STA56120.2022.10018991.

[22] Ghemari, Z., Belkhiri, S., Morakchi, M. R. & Saad, S. Enhancing the piezoelectric accelerometer for effective monitoring and diagnosis of engineering structures. *Rom. J. Acoust. Vib.* **21**, 12–19 (2024).

[23] D’Alessandro, A., Scudero, S. & Vitale, G. A review of the capacitive MEMS for seismology. *Sensors (Switzerland)* **19**, 1–22 (2019). 10.3390/s19143093 PMC6679216 31336990

[24] Akhter, R. & Sofi, S. A. Precision agriculture using IoT data analytics and machine learning. *J. King Saud Univ. Comput. Inf. Sci.*10.1016/j.jksuci.2021.05.013 (2021).

[25] Zhao, Y. et al. Wireless IoT motion-recognition rings and a paper keyboard. *IEEE Access* **7**, 44514–44524 (2019).

[26] Kamble, A. & Bhutad, S. IOT based patient health monitoring system with nested cloud security. In: *2018 4th International Conference on Computing Communication and Automation (ICCCA)*, 1–5 (2018) 10.1109/CCAA.2018.8777691.

[27] Sharma, N. et al. A smart ontology-based IoT framework for remote patient monitoring. *Biomed. Signal Process. Control* **68**, 102717 (2021).

[28] Djeffal, S., Ghoul, A., Morakchi, M. R., Mahfoudi, C. & Belkedari, M. Optimized computer torque control and dynamic model of a spatial single section continuum robot. *Results Control Optim.* **12**, 100264 (2023).

[29] Nistler, J. R. & Selekwa, M. F. Gravity compensation in accelerometer measurements for robot navigation on inclined surfaces. *Procedia Comput. Sci.* **6**, 413–418 (2011).

[30] Gururajan, S., Mitchell, K. & Ebel, W. Flights of a multirotor uas with structural faults: Failures on composite propeller(s). *Data* **4**, 1–12 (2019).

[31] Morakchi, M. R., Defdaf, M., Ghemari, Z. & Djeffal, S. Prototype of an affordable continuum robot-based IoT accelerometer and its kinematic modeling. In: *2022 International Conference of Advanced Technology in Electronic and Electrical Engineering (ICATEEE 1_6)* (IEEE, 2022). 10.1109/ICATEEE57445.2022.10093759.

[32] Devi, C. & Gowri, S. An automatic smart phone with IoT based accident detection and alerting system. In: *Proceeding of the 5th International Conference on Electronics, Communication and Aerospace Technology ICECA 2021*, 426–432 (2021). 10.1109/ICECA52323.2021.9676093.

[33] Morakchi, R., Ghemari, Z. & Defdaf, M. Refinement of capacitive accelerometer performance and its applications in the Internet of Things (IoT). (University of M’sila, 2024).

[34] Morakchi, R., Ghemari, Z. & Defdaf, M. The optimal accelerometer parameter integrated into the mathematical model for avoiding detection errors. In: *1st International Conference on Engineering and Applied Natural Sciences on 10–13 May in 2022 at Konya/Turkey*. 6–12.

